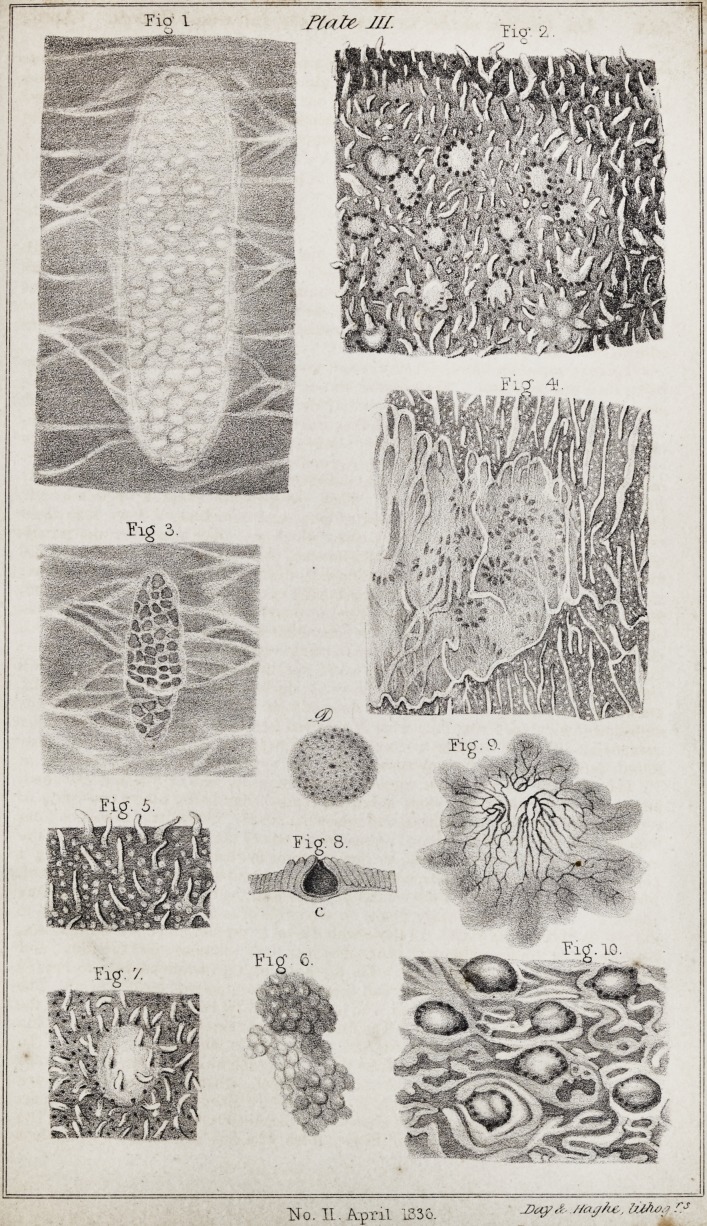# On the Minute Structure of the Intestinal Glands

**Published:** 1836-04

**Authors:** 


### Art. XII.—*De Glandularum Intestinalium Structura Penitiori. Commentatio Anatomica scripsit* Dr. Ludovicus Boehm. *Cum II. Tabulis æri incisis.*—Berolini, 1835. 4to. pp. 54.

*On the Minute Structure of the Intestinal Glands.* By Dr. Ludovicus Boehm.—Berlin, 1835.

The present thesis has substituted a minute and careful anatomical description and arrangement, for our hitherto incomplete and inaccurate knowledge of the glandular apparatus of the intestinal canal. After a close examination of their general anatomy, and a microscopic investigation of their minute structure, Dr. Boehm has classified the various intestinal glands, and has accurately distinguished many that have hitherto been confounded. This is particularly the case in his distinction between the glandulæ solitariæ and those of Brunner, the structure and situation of which are as different as is their tendency to become diseased. The various sources which have contributed to a diversity of opinion among anatomists who have attended to the intestinal canal, are exemplified by a comparison of the different forms of the glandular structures in different animals; and, to avoid those errors which have frequently arisen from the examination of structures particularly liable to morbid affections, in individuals who have died from disease, and whence incorrect inferences have been drawn, Dr. Boehm selected the intestines of suicides, or of those who had met with sudden death.

In our last Number we adverted briefly to the investigations of Dr. Boehm; but we regard them as of sufficient importance to demand from us a more detailed exposition; and accordingly we present our readers, in the following pages, with a condensed translation of all the more important passages in the work, and give in a plate (Plate III.) the more valuable of his illustrations. In executing our task, we shall not interrupt the descriptions by any remark or criticism of our own; we shall only observe, in this place, that Dr. Boehm's speculations respecting the functions of the various glands are not quite satisfactory. A comparison of the secretions of mucous membranes, generally, would have tended much to confirm or disprove the theory which is offered as to the uses of the corpuscles of the glands of Peyer. This comparison has been but partially instituted. The application also of his researches into the anatomy and physiology of these glands, to their pathology, is very limited, and presents nothing new, excepting as it regards the diseases of the glands of Peyer.

*On the general Anatomy of the Glands of Peyer.* In the small intestine of the mammalia, sometimes throughout its whole course, at others in a more limited extent, are seen insulated spots, generally of an oval form, and containing certain corpuscles; the intestinal parietes at these places having lost their transparency, and become thickened. The size of these spots, or glands of Peyer, increases until they attain their maximum at the junction of the cæcum with the ilium. With but few exceptions, they are situated opposite the insertion of the mesentery. These glands are composed of crowded, but distinct and hollow corpuscles; one extremity of which is attached to the mucous membrane, the other projects into the canal. This little eminence is of a remarkably white colour, and circumscribed; in man, either round or flat. On slitting open the intestine, and examining Peyer's glands, both here and in the other parts of the mucous membrane, are found the mucous follicles or crypts of Lieberkühn. But the most conspicuous objects are elevations of a milk-white colour, occupying in great number the glandular apparatus, scarcely a line in diameter, situated in the submucous tissue, and covered by the mucous membrane. On removing the mucous membrane, together with these bodies, numerous indentations remain in the submucous tissue. Each corpuscle consists of a thin and pellucid capsule, which appears white, in consequence of a similarly coloured fluid within it. The cavity and its contents are best seen by placing a portion of the gland in water, and examining it with the point of a needle, beneath a microscope. A milky fluid, which renders the water turbid, escapes, and at first obscures the view of the capsule. When it is empty, the round cavity may be observed. It is as broad, but not so deep, as the projecting corpuscle, and is entirely destitute of cells. Occasionally the capsule is quite empty; in which case it is examined with much more difficulty, on account of its flaccidity, and the absence of its distinguishing colour. The corpuscles are often found empty after death from acute inflammation or violent fevers. In those whose death has resulted from chronic disease, these bodies are so changed or destroyed, that it can be no matter of surprise that, among those who have examined them, there should be a difference of opinion. Two laminæ constitute this capsule, and these I have only been able to distinguish in man. It sometimes happens, whilst dissecting under a microscope, that a thin epithelium is removed from the surface of a corpuscle containing fluid, without either an opening being made into the cavity or escape being given to its contents. This epithelium, as it extends to the parts surrounding the prominent capsules, must belong to the mucous membrane. In some morbid changes, the epithelium has been more readily separable.

*The Crown of Tubes.* The corpuscles constituting the glands of Peyer in mammalia are surrounded with a remarkable crown of small tubes. On examining the margin of each prominent corpuscle, a series of circular apertures may be observed, either surrounding it or situated upon it. In man these apertures are rarely more than ten in number; in the horse and sheep I have counted more than forty. From within each aperture, white processes extend to the mucous membrane. Sometimes these apertures are round, at others oblong, and radiated by processes extending from a central corpuscle; a form which is found to exist in newly-born children. Any one unaccustomed to microscopic investigations will best detect these apertures by extending a portion of healthy mucous membrane upon a black surface. What are the uses of these apertures? It seems little rational to suppose that a cavity which is not cellular should be supplied by several excretory ducts. Pressure upon the capsule never caused the escape of its contents by these apertures. No foramina could be detected within the cavity of the corpuscle. On removing a single corpuscle, (which I could effect from the mucous membrane of the horse or sheep,) the circle of apertures did not adhere to the mucous membrane, but remained attached to the separated corpuscle. The openings were then found to pass into small tubes, the processes already mentioned being situated between them. The form of these tubes resembles that of the crypts of Lieberkühn, except that their apertures are sometimes round, at others oblong.

*Corpuscular Sheaths.* The presence or absence of sheaths has the greatest influence in modifying the form of Peyer's glands. In man, these glands appear somewhat raised above the surrounding mucous membrane; in all other mammalia, they are surrounded by a species of annular fossa, as the vaginal papillæ of the tongue. These sheaths are proportioned to the prominence of the glands, and in the ox and horse entirely conceal the glandular apparatus. This structure has given rise to the mistaken notion, that in such cases the glands themselves do not exist. Not only the corpuscles, but also the circle of tubes surrounding the corpuscles, are protected by sheaths; a structure which becomes evident by slightly stretching the mucous membrane, and examining it with a lens. These sheaths probably defend the parts which they enclose.

*The Course of the Blood-vessels.* An extremely subdivided network of blood-vessels, passing from the submucous tunic, surrounds the whole of each corpuscle, so that a successful injection renders them of a deep red colour. In the normal state, the glands of Peyer are not more vascular than the other parts of the intestine; but no portion of it is so ready to receive an additional supply of blood or to form new vessels. Hence the greater redness of the glands in congestion or inflammation of the intestines, and hence their peculiar tendency to disease. It has been commonly said that the corpuscles of Peyer's glands do not differ from simple follicles, and that they have a central aperture. By no mode of investigation have I been able to assure myself of this; unless a central depression, which is very rarely to be seen, and from which no secreted matter can be pressed out, is so to be considered. The circle of foramina already mentioned, and which may be easily detected by a simple lens, has probably been mistaken for an aperture in the corpuscle. The collapsed state of the corpuscles which is seen after death from some diseases may have contributed to the same error; and not unfrequently in such cases a true opening is formed in the central capsule, and this may be so extended as to terminate in its complete destruction. Dark points, probably of a melanotic nature, are often seen in great abundance within the pellucid capsule.

*Contents of the Corpuscles.* Peyer's glands are generally supposed to secrete mucus. The small quantity which can be collected prevents a chemical examination of their peculiar fluid. Certain differences are discoverable by a microscope between this fluid and mucus. Mucus consists mainly of a viscid, pellucid fluid, in which whitish flocculi are suspended. The globules are only contained in these flocculi: according to Weber, the largest are not equal in size to those of the blood, the least are about one half. The fluid of the corpuscles has no pellucid portion, but appears thickish, whitish, not viscid, and mixed with a portion of water: it consists of innumerable round but not regularly formed globules, conglomerated together, and which swim when thrown into water. They are much less in birds than in man and the mammalia; but they are not always of the same size in the same animal: some are less, others of the same size, and others larger, than the globules of the blood. I have found their diameter of English lines,

| | | | | | |
|---|---|---|---|---|---|
| In the rabbit, .. | 0.0022 | 0.0024 | 0.0030 | 0.0032 | 0.0037 |
| In the ox, .... | 0.0020 | 0.0023 | 0.0025 | 0.0029 | 0.0036 |
| In the goose, .. | 0.0015 | 0.0017 | 0.0018 | 0.0021. | |

These globules want both the regular form and the nucleus of those of the blood. Thus, there appears to be a difference between mucus and the contents of the corpuscles. If, however, the innumerable globules are to be considered as mucous granules, it may perhaps be inferred that these cavities add to a pellucid and viscid secretion of the mucous membrane, an abundance of globules, which appear in it as whitish flocculi.

*Glands of Peyer in Man.* Their average number is twenty, the end of the ilium being their principal seat. They extend throughout this intestine, isolated from one another; and, if any exist in the jejunum, the intervals between them are much increased, and the corpuscles of which they consist are less crowded, and with difficulty recognized. Their situation is opposite the insertion of the mesentery: they consist of plexuses, generally oval, rarely round or angular; their long diameter corresponding to the course of the intestine, and their circumference limited by a scarcely prominent margin. (Pl. III. Fig. 1.) They are generally about a finger's length, but in the commencement scarcely half as long, and sometimes I have seen them extending a foot. The valvulæ conniventes, which are more numerous and prominent in the commencement of the small intestine, pass over the plexuses which are found in the jejunum, but do not interfere with those which are found in the ilium; so that it happens that the valves are occasionally twelve times or oftener interrupted. But more frequently the valves, intercepted in the glandular portion, are again connected by densely congregated villi. The accumulated corpuscles present an unequal, elevated, and glandular appearance, as if many vesicles were placed side by side; but undistinguishable separately by the naked eye, unless they have attained their full size. These eminences, arising from the even mucous membrane between the villi, are not surrounded by sheaths, nor are there any mucous crypts within foveolæ; circumstances widely differing from what is observed in the glandular apparatus of other mammalia. Viewed beneath a simple microscope, most of the corpuscles appear round, but not shaped after any certain rule, and appear as they are represented in Pl. III. Fig. 2. Beside these round corpuscles, some are narrow and extended, others curved or semilunar; some subdivided into many parts, others consisting of two corpuscles in close apposition, and somewhat raised. Sometimes processes from their margin give them a radiated appearance; others are found which occupy the same place with one or more villi, or proceed from the back or top of these villi; a peculiar form of Peyer's glands, which I have only found universal in one individual. Most rarely have I detected the central depression in the corpuscles, and never any aperture. The remarks already made on the circle of small openings apply also to the glands of Peyer in man. In those who die of fevers and other general diseases, the corpuscles are found empty: hence they lose their whiteness and turgidity, and become transparent and collapsed. On this account, my investigations into Peyer's glands have been made on suicides and those who have met with sudden death. The intestinal ulcerations which are met with in abdominal typhus do not originate in the glands or corpuscles themselves. The first and most evident change (as Carus has observed), consists in an exudation which takes place in the submucous tissue. To examine this exudation, that part of the ilium where the ulceration has not yet commenced should be selected. In the normal condition, when the mucous membrane is cautiously removed, the submucous tunic around the glands is found more compact and more intimately connected than elsewhere to the mucous and muscular coats. The submucous tunic around a gland of Peyer, which, in a case of abdominal typhus had

*Plate III.*

Fig 1.

Fig. 2.

Fig. 3.

Fig. 4.

D

Fig. 9.

Fig. 5.

Fig. 8.

c

Fig. 7.

Fig. 6.

Fig. 10.

*Day & Haghe, lithog.rs*

been apparently but a short time changed, when examined in the same manner, was evidently destitute of cellular structure in that part, and exhibited a solid, whitish, grey mass, which, when subjected to a compound microscope, retained no vestige of the organic structure, which we should have expected, but was evidently interwoven with fibres of cellular tissue. This deposit, which is often so abundant as to acquire an elevation of several lines, if formed in larger quantity, elevates the glandular apparatus, whilst the corpuscles themselves are scarcely changed, so that its margin is converted into a broad border. The solidity, density, and peculiar colour of this effused matter constituted a difference between it and that to which the swelling of common inflammation is ascribed. Thus, the true inflammation of the intestine is not to be considered as primary, but as consequent on the distention and irritation of the mucous membrane of the corpuscles covering the exudation: the continuance of this cause leads to ulceration of the corpuscles and glands themselves. The mucous membrane being destroyed, the bottom of the ulcer is formed by the exuded matter, which is hence lardaceous, hard, often rough, unequal, furrowed, and surrounded by callous margins, often of a peculiar yellow colour. Mercurial injections frequently pass through the enlarged mouths of the sanguiferous vessels; hence it is not surprising that hemorrhages sometimes occur. I do not contend that in abdominal typhus all the ulcers are formed in this manner: such would be contrary to the experience of others, and to what I myself have observed in some instances.

*Peyer's Glands in Infants, and the Evolution of the Intestinal Villi.* The extremely delicate mucous membrane of newly-born children, when spread upon a black surface, appears like a most delicate network, the spots in which are formed by the glands of Lieberkühn. The villi are not always uniform. Although they are often of the same form as in the adult, still in some instances, instead of single cylindrical villi, I have found small folds extending to the same limits as the circular muscular fibres. The glands of Peyer may be seen with equal facility as in the adult, by examining a portion of small intestine towards the light. Their number is the same: their size proportional to the smaller circumference of the intestine, and about equal to that of a bean; their margin is circumscribed. Aided by a simple microscope, the greater part of the glandular apparatus is seen to be covered with crowded folds, flexed in various directions, one of which always surrounding the gland in its sinuous course, constitutes, as it were, its margin, and is more elevated than the rest. (Pl. III. Fig. 3.) Billard has considered these folds to consist of glands partially evolved, with glands lying hid beneath them; and that subsequently, as the glands themselves arise, the folds diminish, and eventually disappear. But, in newly-born children, corpuscles are distinguishable between the folds. The real nature of these folds will be evident, on a comparison with the villi of the other part of the mucous membrane. These villi in infants form broad folds, generally running in the direction of the valvulæ conniventes, and sometimes coalescing. There can be no doubt that these folds, in advancing life, pass into the state of villi. What opposes the conclusion that the same metamorphosis happens to those folds which occupy the glandular apparatus? The surface of Peyer's glands in infants is beset with sinuous folds, not with villi; the latter occupy the place of the former in adults. The folds in infants are much more frequent in Peyer's glands than in other parts of the mucous membrane; and, in adults, the villi are far more numerous at the same situation: but sometimes, by the adhesion of several of these villi, their primitive form is lost. The relationship between the folds and villi is also confirmed by the fact, that in many animals, for instance the ox and sheep, the folds in the course of the jejunum are gradually diffused, until at length in the ilium villi alone exist. And, in man, the mucous membrane of the duodenum is covered with reticulated folds, which are gradually more and more formed into single lamellæ, and at length pass into villi. Reptiles and fishes are without villi, and in their stead is a large number of folds.

Although the corpuscles of Peyer's glands in infants are smaller and less defined, they are easily separated. In adults, they arise from the even surface of the mucous membrane; in infants, they exhibit the same general structure as in the mammalia. In the surface of the glands, many broad pits are observed, surrounded by folds, in which the corpuscles are contained. (Pl. III. Fig. 4.) The corpuscles are small, white, and give off radiating processes, separated by depressions. In early life, these processes almost unite in the central corpuscle; but, as this rises and increases, the rays become less; and at length, the corpuscle having attained its full size, they become so small as almost to escape observation. They constitute in adults the dissepiments of the crown of tubes. As age advances, those depressions, in which, in the newly-born, the corpuscles are contained, are gradually flattened, and at length disappear; so that the individual corpuscles arise from the otherwise even glandular surface, like little eminences between the bases of the villi.

It is unnecessary to dwell on that part of the present Essay which is devoted to the comparative anatomy of Peyer's glands in the mammalia, more than to notice the structure, which under various modifications has contributed to confirm the notion that there are apertures in the corpuscles of these glands in the human subject. In the horse, several corpuscles are sometimes contained in one sheath, like eggs in a nest. The aperture of the sheath has been mistaken for an aperture in the corpuscles themselves, which have been overlooked. The same is the case in the ox, the sheaths being very deep, in the bottom of which are situated the corpuscles. When these bodies are examined, the crown of tubes and other distinctive characteristics of the corpuscles are readily distinguished. The usual structure of Peyer's glands is substituted in birds by crowded and peculiarly formed villi. These villi form, by their junction, a plexus, which contains cells passing deeply into the mucous membrane. These cells may be compared to the sheaths of the corpuscles in the mammalia. There are no corpuscles within these cells; but, in their place, foramina, which descend to sacculi that are prominent on the outer surface of the mucous membrane. Excretory ducts, which are not found in mammalia, here exist, and on pressure give escape to a whitish fluid. The bases of the sacculi, surrounded by the vascular tissue, sometimes penetrate the muscular coat, and may be seen through the serous membrane. The fluid contains innumerable globules; generally round, but less in size than in the mammalia.

*Cryptæ minimæ, Folliculi minimi, seu Glandulæ Lieberkuhnii.* Innumerable foramina beset the mucous membrane of the whole intestine: those in the large must be considered separately from those in the small intestine; not on account of their different structure, but because the former are larger and more easily recognized; the latter so small, that, in a healthy intestine, they can only be seen by the aid of a magnifying power. I shall first describe the latter. Lieberkühn thus speaks of them. "The surface of the intestine is occupied by villi, the bases of which are separated somewhat from one another. On the surface of the intestine, parallel to the bases of the villi, may be detected, by a careful examination, numerous open mouths of follicles, and in the bottom of these, whitish bodies are situated." Lieberkühn considered these corpuscles as glands. Haller was of the same opinion. Others have not only disputed their nature, but their existence; and among the latter were Cuvier and Billard. Rudolphi contended that they were not glands, but subservient to the function of absorption. After the most careful examination of these organs, both in man and animals, both in the healthy and diseased state, I am convinced that they are not foramina perforating the mucous membrane, but secreting cavities, situated in a simple recess of the mucous membrane, and to be considered as simple glands. In order to detect these crypts, the mucous membrane of infants should be selected: and, for a comparative experiment, the delicate membrane of other mammalia. Being stretched upon a black surface, and immersed in water, the intervals between the villi are seen studded by innumerable black apertures, which, in the fœtus and newly-born child, are so abundant as to be almost in contact. The intervals increase in the adult, so that they occupy more space than the apertures. Lieberkühn probably rather overstated the fact, in saying that each villus was surrounded by eight apertures. Every aperture leads to a simple follicle, the depth of which is in proportion to the thickness of the mucous membrane. The follicles are situated in the mucous membrane itself, though they do not project beyond its external surface. In this character, they appear to differ from all the other follicles, and particularly from those of the large intestine: for those which occupy the end of the colon project like long tubes from the external surface of the mucous membrane; those, however, of the ascending colon are more like the crypts of the small intestine. If a difficulty is found in ascertaining the state of the crypt as described, this must be attributed to their minuteness; and, in order to preserve the very thin membrane at their base, the mucous membrane must be most carefully removed, lest, by tearing it from the subjacent membrane, foramina should be artificially produced. I have found this follicular structure most distinct in the mucous membrane of the wood pigeon. Are the round whitish bodies in the bottom of these follicles to be considered glands, as Lieberkühn supposed? They are generally absent in healthy crypts. I have noticed them after death from inflammations, particularly of the intestines, (Pl. III. fig. 5;) in idiopathic nervous fever, in which, although there was no ulceration of the mucous membrane, a sub-inflammatory condition of it existed: also in cases of death from general dropsy, and in scrofulous infants. (Pl. III. Fig. 4.) Hence I conclude, that this whitish substance in the crypts is a retained secretion. The chief arguments in favour of this idea are, the absence of these bodies in health; their presence in disease; their greater abundance and size when and where diseased action is most manifest; the fact, that sometimes a crypt is completely filled with this whitish substance, and that it is occasionally met with between the villi. Is it not a farther presumption adverse to their glandular nature, that, although Lieberkühn was so successful an injector, he never mentions their vascularity?

*Glandulæ Conglomeratæ seu Brunneri.* Different glands, and particularly the glandulæ solitariæ, have been confounded with those of Brunner. Brunner's glands are situated principally in the duodenum. To expose them, it is necessary to separate the serous and muscular coats; when, next to the lower sphincter of the pylorus, in the submucous tissue, a continuous layer of whitish glands, surrounding the whole duodenum, is exposed; their number diminishes towards the end of the duodenum, or commencement of the jejunum, in which situation they are no longer seen. Their form is angular; their size, unless diseased, scarcely that of a hemp-seed; each one is contained in cellular tissue; either end is inserted into the mucous membrane, whence I have been frequently able to draw out their excretory duct. The glands consist of lobules, the ducts of which open into a common excretory duct, and each lobule appears to consist of six hundred acini, so that the whole gland very much resembles a raceme. Hence the glands of Brunner belong to the conglomerate class.

*Glandulæ Solitariæ.* These are scattered throughout the whole tract of the small intestine, including also the valves. Fewer in its commencement, their number increases in its course, so that, when tumefied by any irritation, they occupy the whole inner surface of the end of the ilium. They are not found beyond the valve of the colon. Elevated by their contents, they may be readily felt. They are scarcely, if at all, prominent on the external surface of the mucous membrane. When magnified, they appear like round vesicles, with a circle of foramina, similar to those of Peyer's glands. The peculiarity of their structure is represented in Pl. III. Fig. 7. When cut, their cavity is found simple, round, and filled with a white substance. These glands are formed, and increase in infants, as the glands of Peyer. They have been confounded with those of Brunner. In almost all the affections of the mucous membrane, these glands are implicated; they are simultaneously affected with the glands of Peyer. The supposed pustules, to which some have ascribed the ulceration in abdominal typhus, are very probably the solitary glands tumified, and, in a careless examination, readily mistaken for pustules; and the more so, if the top has been ulcerated, and an opening is formed. I have never seen the glands of Brunner diseased in typhus. The fluid in the solitary glands contains innumerable globules, such as are found in the corpuscles of Peyer's glands.

Two kinds of glands are found in the large intestine.

*Glandulæ Minores Simplices seu Tubulatæ.* An innumerable quantity of pores, very evident by the aid of a simple lens, may be seen on the inner surface of the large intestine, which open by a rounded aperture through the mucous membrane. These glands (and, if the crypts of Lieberkühn are so called, the name is not less appropriate here,) increase in size as they approach the end of the rectum. A careful examination of the cæcum reveals nothing but apertures. There is no elevation of the outer surface of its mucous membrane; and hence, as the crypts of the small intestine, they are with difficulty examined. But in the transverse colon, the apertures are extended as sheaths; and, on cutting the extended mucous membrane at a right angle, the parietes of short follicles are brought into view. These same follicles, in the end of the rectum, are extended as long tubes, evident to the naked eye, and investing the whole external surface of the mucous membrane; constituting a peculiar layer between the mucous and muscular coats; the tubes being erect, parallel, and densely crowded. These tubular follicles open into the intestine, their closed extremity being inserted into the submucous tissue. The peculiarly thick and tenacious mucus of the large intestine is doubtless formed by these glands, which are also the source of the mucous, if not of the bloody secretion accompanying piles, and perhaps the true seat of dysentery. In the secreted fluid, I have never detected the round globules, which are so abundantly formed in Peyer's glands.

*Glandulæ Majores Simplices.* Simple and large follicles are distributed over the whole inner surface of the large intestine, which have been called solitary. They are more abundant where a large quantity of mucus is required, as in the cæcum and processus vermicularis; in the corner of the former of which fæces are liable to be retained. These large glands are less abundant in the colon, more frequent again in the rectum. They consist of a simple cavity, which is contained in a capsule inserted in the mucous membrane. They have been incorrectly confounded with Brunner's and the solitary glands. Their distinction from Brunner's glands will be seen by comparing Pl. III. Fig. 6, with Pl. III. Fig. 8. c. The cavity terminates in a short duct which opens on the surface of the mucous membrane. This membrane at the situation of the gland is slightly elevated, (Pl. III. Fig. 8. d.,) the surface of which elevation, not less than the other parts of the membrane, is perforated by the numerous apertures of the glandulæ minores, which closely surround the capsule. The capsule is equally prominent at the outer surface, where it is surrounded by the submucous tissue, the very minute vessels of which pass to it, and, interwoven with it, constitute an elegant network, which is subservient to the secretion of mucus within its cavity. (Pl. III. Fig. 9.)

### *Explanation of Plate III.*

Fig. 1. A gland of Peyer, taken from the end of the ilium of a man who died suddenly. In order to render the gland more evident, the transparent mucous membrane to which it adheres, separated from the other tunics, is stretched over a black surface.

Fig. 2. The right side of the gland (Fig. 1.) magnified by a simple microscope, shewing the variously formed corpuscles, surrounded by an elegant crown. They are generally round, some are oval. There is no vestige, in the centre of their summit, of an excretory aperture. One or more villi are not unfrequently inserted on the surface of the capsule. The union of two corpuscles sometimes constitutes a single elevation. One alone, and this is extremely rare, exhibits a central depression, resembling an excretory duct. In this gland, as in the other parts of the mucous membrane, are seen the crypts of Lieberkühn and the villi.

Fig. 3. A gland of Peyer, taken from an infant of seven months. The surface is covered with tortuous and connected folds, which disappear in advancing years.

Fig. 4. A magnified representation of the right side of the gland (Fig. 3.) The little corpuscular capsules, deeply seated, give off from their margins long processes or rays, resembling stars. A continuous and tortuous fold limits and constitutes the border of the gland. Similar folds occupy the whole gland and the mucous membrane. The crypts of Lieberkühn appear as white points, their cavities being occupied by their thick secretion.

Fig. 5. The mucous coat of the small intestines, as found altered in inflammations and acute fevers. The glands of Lieberkühn are represented as they are found turgid and distended by a tenacious white secretion, scarcely soluble in water. The portion of membrane here represented is that of an adult cut off by abdominal typhus, and is magnified eighty times.

Fig. 6. A conglomerate or Brunnerian gland, taken from the commencement of the duodenum of a man, and magnified one hundred times. The cellular tunic is removed from the submucous coat in which the gland is concealed, so that three lobules are brought into view. These lobules consist of smaller rounded acini. The excretory duct on its opposite surface is inserted into the mucous membrane.

Fig. 7. A solitary gland, of the same character as those which are met with in the whole tract of the small intestine. The structure is little different from that of the corpuscles of Peyer; the crown of apertures is also present. The villi are so numerous, that they not only occupy the margin, but cover nearly the whole elevation of the capsule.

Fig. 8. Glandulæ majores simplices. These are found everywhere scattered throughout the whole large intestine.

C. One of these glands taken from the human rectum, divided vertically into two halves. It is unusually large; its round cavity and excretory aperture are exhibited. The glandulæ tubulatæ surround the capsule closely to its summit.

D. A gland viewed at its upper part. It elevates the mucous membrane in the form of a little umbilical prominence, the centre of which is occupied by an excretory aperture. The surface is perforated by very numerous openings, which belong to the glandulæ tubulatæ.

Fig. 9. The vascular structure occupying the surface of the prominent glands, the apertures being omitted. The vessels coalesce more and more towards the top, where many of them form covered nooses.

Fig. 10. Part of a gland of Peyer, taken from the jejunum of a sow. Here each corpuscle is surrounded, not by villi, but by tortuous folds, such as exist in the rest of the mucous membrane.

## Figures and Tables

**Fig 1. Fig. 2. Fig 3. Fig 4. Fig. 5. Fig. 6. Fig. 7. Fig. 8. Fig. 9. Fig. 10. f1:**